# High frequency of intron retention and clustered H3K4me3-marked nucleosomes in short first introns of human long non-coding RNAs

**DOI:** 10.1186/s13072-021-00419-2

**Published:** 2021-09-27

**Authors:** Pinki Dey, John S. Mattick

**Affiliations:** School of Biotechnology and Biomolecular Sciences, UNSW Sydney, 2052 Sydney, Australia

**Keywords:** Long non-coding RNAs, Exons, Introns, Nucleosomes, Histone modifications

## Abstract

**Background:**

It is established that protein-coding exons are preferentially localized in nucleosomes. To examine whether the same is true for non-coding exons, we analysed nucleosome occupancy in and adjacent to internal exons in genes encoding long non-coding RNAs (lncRNAs) in human CD4+ T cells and K562 cells.

**Results:**

We confirmed that internal exons in lncRNAs are preferentially associated with nucleosomes, but also observed an elevated signal from H3K4me3-marked nucleosomes in the sequences upstream of these exons. Examination of 200 genomic lncRNA loci chosen at random across all chromosomes showed that high-density regions of H3K4me3-marked nucleosomes, which we term ‘slabs’, are associated with genomic regions exhibiting intron retention. These retained introns occur in over 50% of lncRNAs examined and are mostly first introns with an average length of just 354 bp, compared to the average length of all human introns of 6355 and 7987 bp in mRNAs and lncRNAs, respectively. Removal of short introns from the dataset abrogated the high upstream H3K4me3 signal, confirming that the association of slabs and short lncRNA introns with intron retention holds genome-wide. The high upstream H3K4me3 signal is also associated with alternatively spliced exons, known to be prominent in lncRNAs. This phenomenon was not observed with mRNAs.

**Conclusions:**

There is widespread intron retention and clustered H3K4me3-marked nucleosomes in short first introns of human long non-coding RNAs, which raises intriguing questions about the relationship of IR to lncRNA function and chromatin organization.

**Supplementary Information:**

The online version contains supplementary material available at 10.1186/s13072-021-00419-2.

## Introduction

The genome is composed of protein-coding and non-coding regions collectively transcribed into a large and complex transcriptome that regulates the cellular machinery. The protein-coding component of the human genome (including 5′ and 3′ flanking cis-regulatory sequences in mRNAs) comprises only 2% of the total, whereas the vast majority is differentially transcribed [1, 2] to produce a plethora of small and large non-protein-coding RNAs (ncRNAs) that are expressed intergenically, intronically and antisense with respect to protein-coding genes [3–9].

Chromatin structure and transcription are temporally and functionally related [10–12] and chromatin architecture has a significant impact on gene expression [13–16]. The structural unit of eukaryotic chromatin is the nucleosome wherein approximately 147 base pairs (bp) of genomic DNA is wrapped around an octamer of 4 histones and separated from the neighbouring nucleosomes by a 20–80 bp variable stretch linker DNA [17]. The histones are extensively modified, which transmits epigenetic information during differentiation and development, imposed by enzymes that are likely directed to specific genomic loci by RNA guides [14, 18–20], as a second-derivative of genomic information.

Over the past decade, hundreds of long non-coding RNAs have been shown to play important roles in cell and developmental biology [21–23], as well as in the aetiology of cancer and other diseases [24–27]. These observations suggest that the regulation of multicellular ontogeny cannot be explained solely by the combinatorial control of gene expression by transcription factors, histone modifiers and other widely expressed proteins, but should also include the interaction of regulatory RNAs with them.

Similar to protein-coding genes, many lncRNAs are transcribed by RNA polymerase II and feature a 5′ cap and polyA tail [1, 28]. Some lncRNAs are relatively highly expressed, such as Neat1 and Gomafu, which nucleate specialized subnuclear domains [29–33]. Most are relatively ‘lowly’ expressed, which led to the suspicion that they largely represent transcriptional noise. Undersampling in RNAseq datasets also led to incomplete transcript models. However, high-resolution studies have shown that most lncRNAs are precisely expressed in specific cells and developmental stages, and are therefore not well polled in bulk RNAseq datasets, especially those from complex tissues like brain [34–36]. They have also shown that most lncRNAs show specific subcellular locations, are multiexonic and exhibit extensive alternative splicing [34, 37, 38].

Two decades ago, Trifonov et al. showed that splice sites correlate with nucleosome positions [39, 40]. In 2009 several groups confirmed that nucleosomes are preferentially positioned over protein-coding exons, as well as in non-coding exons in 5′UTRs and some annotated non-coding genes [41–45]. These observations indicate that epigenetic regulation of gene expression is not simply gene-specific but exon-specific, possibly including exon selection for alternative splicing.

We sought to re-visit the matter with the much more extensive lncRNA annotations and nucleosome position/histone modification databases that have appeared over the past decade. We confirmed that lncRNA exons are globally, like coding exons, preferentially located in nucleosomes, but also discovered some unexpected features.

## Methods

Nucleosomal data sets for the human total nucleosome library were obtained from the data produced by Schones et al*.* [46] [NCBI Short Read Archive (SRA) accession number SRP000105] who produced genome-wide nucleosome maps of resting and activated human CD4+ T cells by Solexa high-throughput sequencing of DNA purified by micrococcal nuclease digestion (MNase). Nucleosome libraries were downloaded from https://trace.ncbi.nlm.nih.gov/Traces/sra/?study=SRP00010. As discussed by Schmid et al*.* [47], we extended all uniquely mapped short-read sequences of the nucleosome library to the expected 150 basepair (bp) in the 3′ direction. This is because the raw sequence tags are derived from the ends of the strands rather than over their whole length and signals from the plus and minus strands associated with the same nucleosome are typically ~ 150 bp apart.

The nucleosomal data for the histone-modified nucleosome libraries were obtained from the data generated by Barski et al. [48] [NCBI Short Read Archive (SRA) accession number SRA000206] who generated genome-wide nucleosome maps of 20 histone lysine and arginine methylations from MNase digested CD4+ T cells using Solexa 1G sequencing technology. We downloaded the data from https://dir.nhlbi.nih.gov/papers/lmi/epigenomes/hgtcell.aspx. Both the total and the modified nucleosome libraries were in hg18 genome assembly which has been converted to the hg38 genome assembly by the LiftOver standalone tool from University of California Santa Cruz (UCSC) [49]. The H3K4me3 and H3K36me3 ChIP-seq datasets of the human H1 embryonal stem cell line[50] and IMP-90 foetal lung myofibroblast cell line [51] were obtained from ENCODE with identifiers ENCFF775QSF, ENCFF247BVI, ENCFF669PQL, ENCFF582IQY, ENCFF441KOL,ENCFF694EXK, ENCFF890EPF, ENCFF334FBV, ENCFF969ZSU, ENCFF994SJM, ENCFF391ENY, ENCFF648MBH, ENCFF547BFL, ENCFF308CPT, ENCFF202AKQ. The H3K4me3 datasets for the B cell, T cell, CD4+ T cell and CD8+ T cell types were obtained from ENCODE with identifiers ENCSR939UQD, ENCSR395YXN, ENCSR852FRR, ENCSR796CSH, respectively.

The coordinates for the lncRNA exons were obtained from GENCODE Release 38 (GRCh38.p13) [7] (https://www.gencodegenes.org/human/). We extracted the coordinates of mRNA exons from the hg38 reference human genome by excluding the exons of GENCODE annotated lncRNAs. Hence, the mRNA label used in our study denotes all exons annotated in the human genome except those from lncRNAs. The coordinates of non-overlapping introns were extracted from the exon coordinates by GTFtools [52]. In-house scripts and Bedtools [53] were used to convert the BED coordinates to UCSC wiggle files and to calculate the average nucleosome densities. The average count of tags mapped at each nucleotide position of the first or last 80 bp of exons and 500 bp of the flanking introns were calculated. The density of reads mapping to each nucleotide position was normalized for the number of exons polled and the depth of the nucleosome library.

The coordinates for constitutively spliced (total 135,461) and alternatively spliced (total 91,718) internal exons were obtained from the HEXEvent database, which reports all known splice events based on EST information from the UCSC Genome Browser [54].

The intron retention levels in the RNA seq dataset were estimated using the R module SIRfinder [55] based on gene annotations for the hg38 reference genome. The total RNAseq data from K562 cells for the (retained introns) IR calculation was obtained from the ENCODE portal [56] (https://www.encodeproject.org/) with the identifier ENCSR885DVH. The H3K4me3 and H3K36me3 ChIP-seq dataset from K562 cells was obtained from ENCODE with identifiers ENCSR668LDD and ENCSR000DWB, respectively.

The plots were constructed using R4.0. The scripts for data processing and analysis are deposited at the Github repository (https://github.com/pdey1/IR-H3K4me3-signal).

## Results

**Fig. 1:**
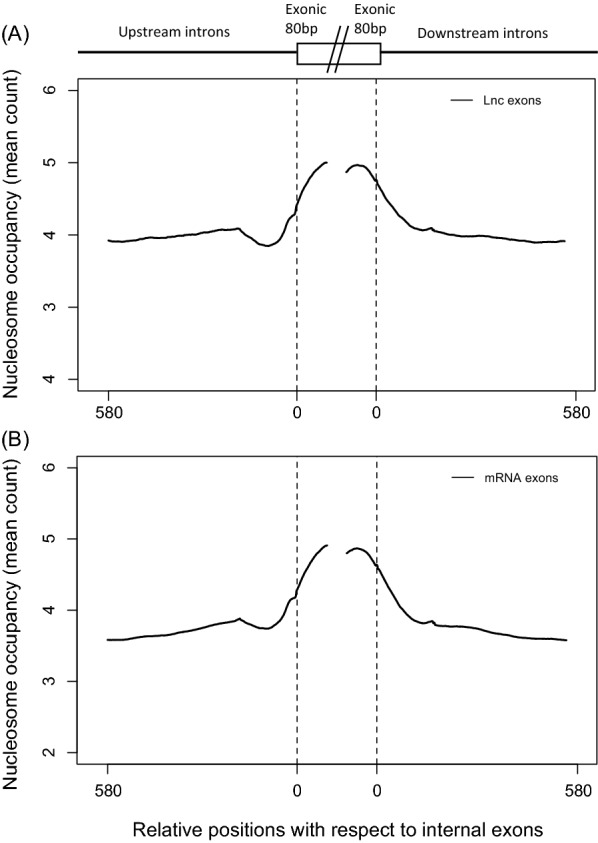
Nucleosome density at **A** internal long non-coding exons and **B** mRNA exons in comparison to flanking introns in CD4+ T cells. The average nucleosome densities over 580 bp upstream and downstream of exons are shown and the central gap indicates the point of discontinuation between ‘upstream’ and ‘downstream’ data series. The nucleosome density is normalized by the number of exons (28,624 and 28,199 upstream and downstream internal lncRNA exons and 141,491 and 136,989 upstream and downstream internal mRNA exons) in each case

We employed total nucleosome deep sequencing libraries and ChIP-Seq histone modification data from CD4+ T cells to analyse nucleosome occupancy upstream and downstream of human lncRNA exons. The upstream and downstream datasets were derived from internal lncRNA exons to remove complications from extended terminal exons or uncertain initiation or polyadenylation sites in transcriptomic datasets. We observed ~ 28% increase in the nucleosome occupancy at lncRNA exons as compared to their flanking introns (Fig. 1A), similar to that observed with exons in protein-coding transcripts (Fig. 1B), indicating that nucleosomes are preferentially and precisely positioned on the long non-coding exons, as they are in as with exons of protein-coding genes.

**Fig. 2:**
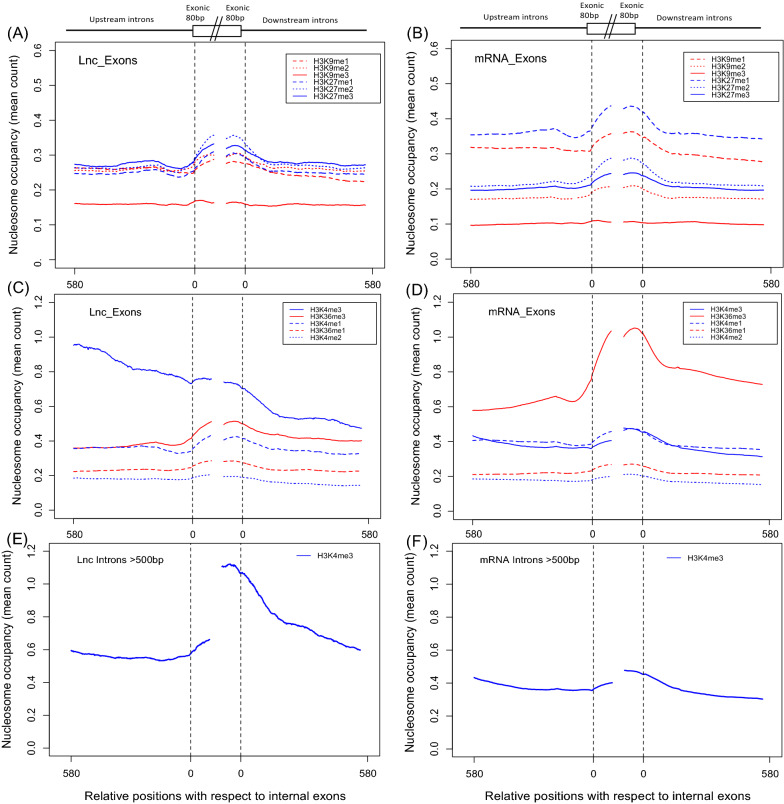
Density of nucleosomes containing different histone H3 modifications in CD4+ T cells on **A**, **C** internal long exons of lncRNA genes; **B**, **D** internal exons of protein-coding genes; **E**, **F** lncRNA and mRNA exons that are separated from adjacent exons by at least 500 bp. The average nucleosome densities over 580 bp upstream and downstream of exons are shown and the middle gap indicates the point of discontinuation between ‘upstream’ and ‘downstream’ data series. The nucleosome density is normalized by the number of exons (28,624 and 28,199 upstream and downstream internal lncRNA exons and 141,491 and 136,989 upstream and downstream internal mRNA exons) in each case

To investigate the role of histone modification on the preferential nucleosome enrichment on lncRNA exons, we analysed the ChIP-Seq data for 12 types of common modifications in the histone modification libraries obtained from CD4+ T cells. We also performed the analysis with mRNA exons and observed significant nucleosomal enrichment in both cases (Fig. 2A–D). We found that nucleosomes are preferentially positioned at exons in most of the histone modification libraries, although some, notably H3K9me3 and H3K4me2 (Fig. 2A–D), did not show significant nucleosome enrichment, which is consistent with previous observations [41].

**Fig. 3:**
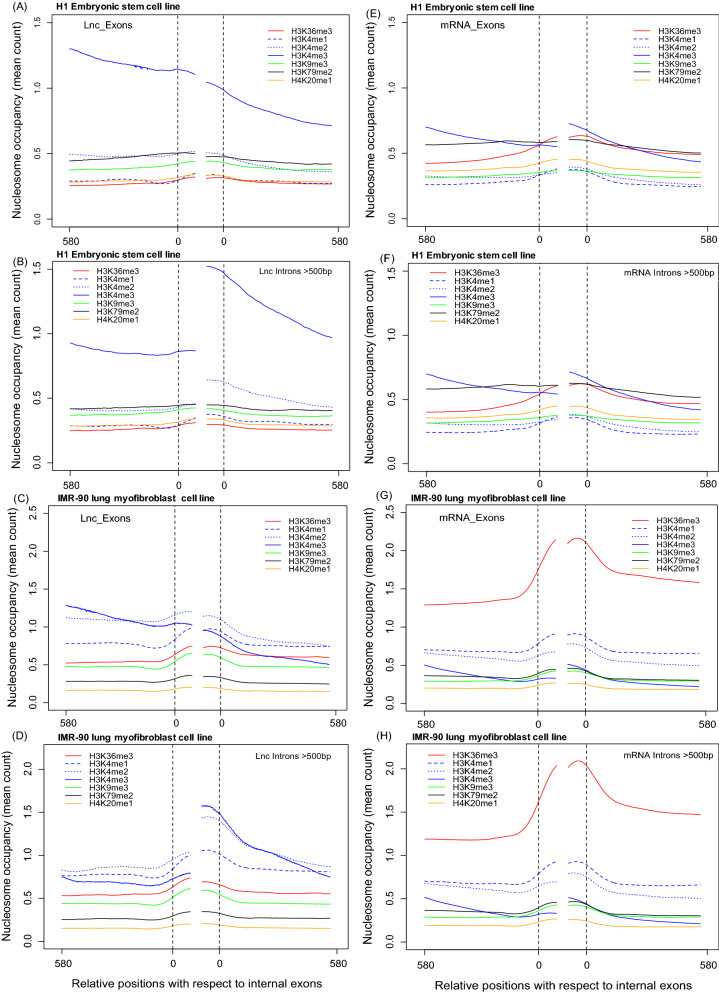
Density of nucleosomes containing different histone modifications on **A**–**D** internal long exons of lncRNA genes, **E**–**H** internal exons of protein-coding genes from two different cell lines, H1 and IMR-90. The nucleosome density was normalized by the number of exons (28,624 and 28,199 upstream and downstream internal lncRNA exons and 141,491 and 136,989 upstream and downstream internal mRNA exons) in each case

There were some notable differences between the plots for mRNA and lncRNA exons. The first is the strong signal for H3K36me3-marked nucleosomes in mRNA exons but not lncRNA exons (Fig. 2C, D), possibly reflecting their (generally) higher expression levels. Second, a relatively higher nucleosomal occupancy of H3K27me1 was observed for protein-coding genes compared to lncRNA genes (Fig. 2A, B), possibly reflecting their positioning on highly transcribed gene bodies [57]. Third, there is high enrichment of H3K4me3-marked nucleosomes upstream of lncRNA exons, which was not observed with mRNA exons. To examine if these associations are also found in other cell lines, we repeated the same analysis with different histone modification libraries from the H1 human embryonal stem cell line and the IMR-90 human foetal lung myofibroblast cell line (Fig. 3).

Again, we found high enrichment of H3K4me3-marked nucleosomes upstream of lncRNA exons in these cell lines. The high upstream H3K4me3 signal in the H1 ES cell line is consistent with the previous finding that H3K4me3 is a prevalent mark near the promoters of genes in human ES cells [58]. We also found that the signal is stronger for lncRNA exons and stronger in ES cells than in the lung cell line. We repeated the analysis on recent H3K4me3 library datasets for four other cell types (B cells, T cells, CD4+ Tcells, CD8+ Tcells) and observed a similar pattern of high enrichment upstream of lncRNA exons (Additional file 1: Fig. S1).

We also found that the elevated upstream H3K4me3 signal was stronger in human alternatively spliced internal exons compared to constitutively spliced internal exons (Additional file 1: Fig. S2A). By contrast, the strong enrichment of H3K36me3-marked nucleosomes in mRNA exons was observed only in the IMR-90 lung cell line and there was no difference in the signal between constitutively and alternatively spliced exons (Additional file 1: Fig. S2B).

We extracted the coordinates of some of the lncRNA intronic regions and examined them in the UCSC Genome browser. Surprisingly, we found that the intronic regions enriched for H3K4me3 signals exhibit both high H3K4me3-marked nucleosome occupancy across the entire intron, along with frequent evidence of intron retention (IR) in lncRNA transcripts.

**Fig. 4:**
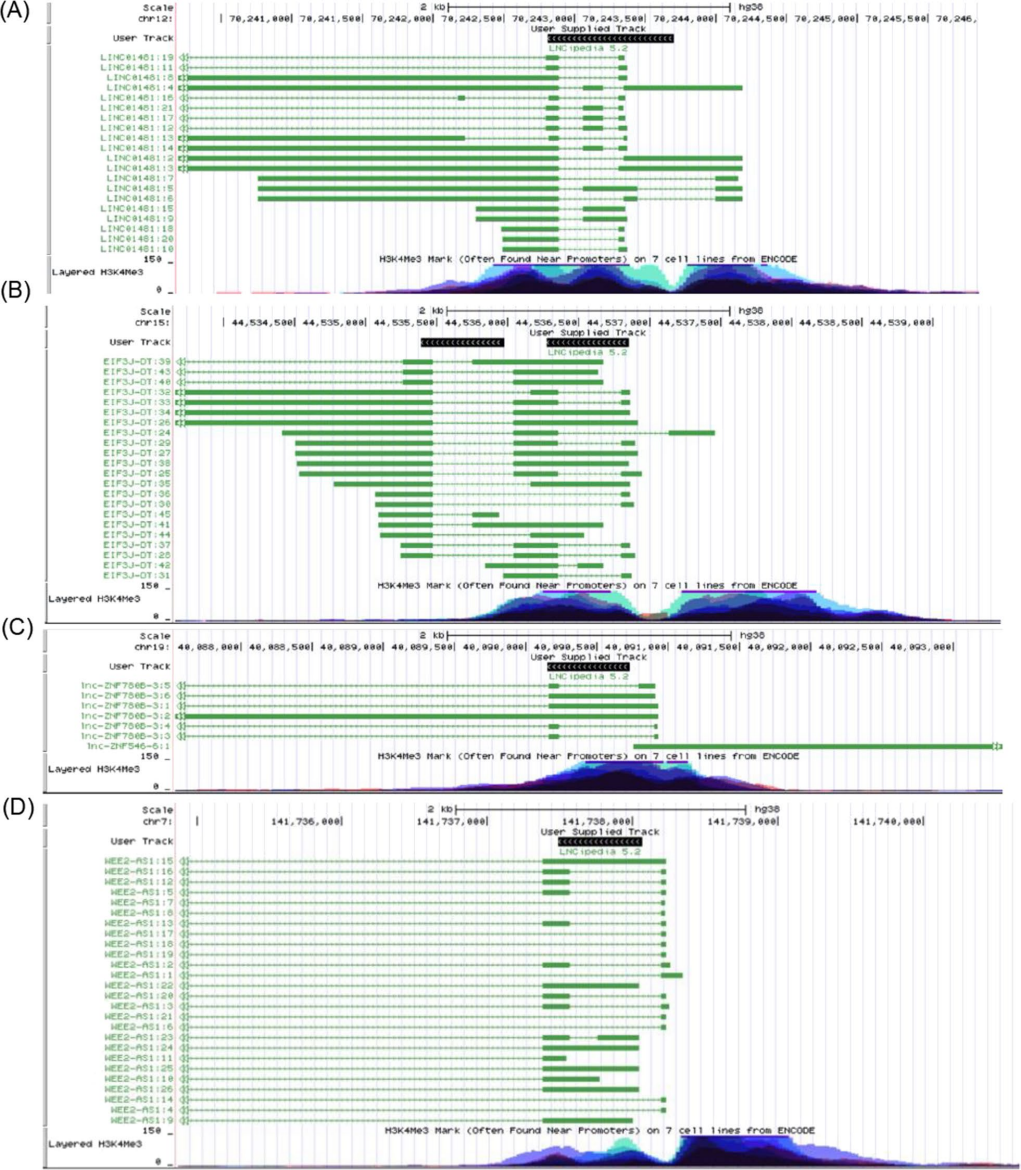
Exemplary screenshots from UCSC genome browser highlighting the correlation between the layered H3K4me3 bar plots from different cell types (blue, cyan, yellow, red, pink bars) and the available long non-coding transcripts (green lines and bars) from LNCipedia [59]. The black bars represent the user-defined regions. The UCSC genome browser links to the tracks are provided in the supplementary material (Additional file 1: Table S1).

We term these extended regions of high H3K4me3 occupancy ‘slabs’ (as opposed to the usual peaks of nucleosome occupancy at specific positions), which extended (and we defined as extending) over at least 1 kb at the maximum of the vertical viewing range of 150 in the bar graphs from seven different cell lines available in the UCSC genome browser layered H3K4me3 track settings. Exemplary UCSC genome browser screenshots highlighting the layered H3K4me3 bar plots and lncRNA transcripts are shown in Fig. 4A–D.

To investigate if intron retention is generally associated with the presence of H3K4me3 slabs, we then examined the occurrence of IR in lncRNA transcripts and slabs (M) of high H3K4me3 expression level across exon–intron–exon blocks in 200 randomly selected intronic regions from lncRNA loci across the genome (Additional file 1: Table S2).

We found that in ~ 70% of the instances the occurrence of IR and M are correlated, i.e., if a slab is present, there is a high coincidence of intron retention in recorded transcripts from the locus, and vice versa (Table1). A Pearson’s Chi-squared test with 95% confidence interval gave a *p*-value of 7e−10, confirming a highly significant correlation between M and IR.

**Table 1:** The occurrence of slabs (M) and retained introns (IR) as observed for user-defined genomic coordinates representing the 200 randomly selected long non-coding intronic regions (Additional file 1: Table S2)

	IR present	IR absent
M present	57	10
M absent	52	81
Median intron length (bp)	192	2181
Average intron size (bp) ± standard error	354 ± 67	5323 ± 841
Frequency of 1st intron	73 (67%)	24 (26%)

We suggest that the incompleteness of the epigenomic and transcriptomic datasets may, in part at least, explain 30% of cases where there was no correlation between the presence of retained introns and H3K4me3 slabs. This suggestion is strengthened by our observation that we observe IR more commonly than slabs, reflecting the fact that there is more transcriptomic data than epigenomic data available.

We also observed that occurrence of intron retention is strongly (67%) associated with the first intron, which explains the asymmetry of the signal in Fig. 2C, and that the length of the retained intron (average ~ 354 bp) is far smaller than the average length of first non-retained introns generally (5323 bp) and all introns (6355 bp and 7897 bp in human protein-coding and non-coding genes, respectively [60].

We therefore repeated the analysis of H3K4me3 occupancy after removing short introns (i.e., intron sequences that overlap an exon within 500 bp; Fig. 2E) and found that the high H3K4me3 signal disappeared, confirming that the H3K4me3 signal slabs are associated with short introns. We also performed the same analysis with the H1 embryonal stem cell and IMR-90 lung epithelial cell line and found the same correlation (Fig. 3B, D). Interestingly, in all cells we also observed an increase in the density of H3K4me3-marked nucleosomes in regions downstream of exons after removing those associated with short introns, suggesting that the latter are depleted for this mark downstream of the retained intron.

By contrast, we did not observe any changes in the nucleosome density analysis after removing the exons separated by short introns in protein-coding genes (Fig. 2F) indicating that the high H3K4me3 signals associated with short first introns are a specific characteristic of lncRNA genes. Removal of short introns made no discernible difference to the distribution of nucleosomes containing histones with the other polled marks in either the lncRNA or mRNA exonic datasets (data not shown).

To examine whether it is primarily the first intron—as indicated by our straw poll of 200 lncRNA loci—that is the source of the high upstream exonic signal in the H3K4me3 nucleosome library, we repeated the H3K4me3 nucleosome density analysis after removing the first and second lncRNA exons (Additional file 1: Fig. S3), which eliminated the upstream elevated signal, confirming the genome-wide association between slabs and first intron retention. This also suggests that short first introns are a general feature of lncRNA genes, in contrast to mRNA genes.

We investigated the genome-wide association between short first introns of lncRNA genes and H3K4me3 nucleosome density by analysing the H3K4me3 nucleosomal density plots of the first lncRNA introns with length less than 500 bp and first lncRNA introns with length greater than 500 bp. We confirmed that the first lncRNA introns with lengths less than 500 bp have a higher nucleosomal density than introns with lengths greater than 500 bp (Additional file 1: Fig. S4). We also found that this is a general feature of lncRNA genes as we did not make similar observations for protein-coding genes. Moreover, we observed that the first mRNA exons less than 500 bp showed a relatively lower nucleosomal density than introns with lengths greater than 500 bp (Additional file 1: Fig. S3B). Our observations are also consistent with the recent report of the relatively short lengths of first introns in lncRNA genes compared to their protein-coding counterparts and the impact of splicing efficiency on intron length, especially that of the first intron [61].

In addition, we calculated the nucleosomal enrichment at lncRNA exons for H3K4me3 histone-modified library by dividing the exonic data into two groups based on the GC content of the exons, since GC content has been linked to gene expression level in mammalian cells [62–64], suggesting a link between chromatin architecture and gene expression. The relative enrichment is significant for both lncRNA and mRNA of exons irrespective their GC content, although the overall nucleosomal occupancy for both lncRNA and mRNA exons varies with respect to the GC content (Additional file 1: Fig. S5). In addition, there is no significant difference in the relative nucleosomal enrichment on both the lncRNA and mRNA exons for RNA PolII Chip Seq data (Additional file 1: Fig. S6), which indicates that the high H3K4me3 signal observed on lncRNA exons is independent of gene expression.

To investigate the correlation, as distinct from the general association, between lncRNA transcripts and slabs, we compared the transcript and H3K4me3-modified nucleosomal datasets from the same cell line, K562 from ENCODE. First we reanalyzed the nucleosome density for the GENCODE lncRNA exons against the H3K4me3 and H3K36me3 nucleosome libraries of the K562 cell line and found the same correlation as seen with the CD4+ T cells, (Additional file 1: Fig. S7). We then calculated the intron retention levels in the RNAseq dataset of K562 cell line using the R module SIRfinder. We extracted the coordinates of the retained and non-retained introns on the basis of SIRatio (if SIRatio > 0, we considered the introns to be retained and if SIRatio = 0, we considered them to be non-retained).

**Fig. 5:**
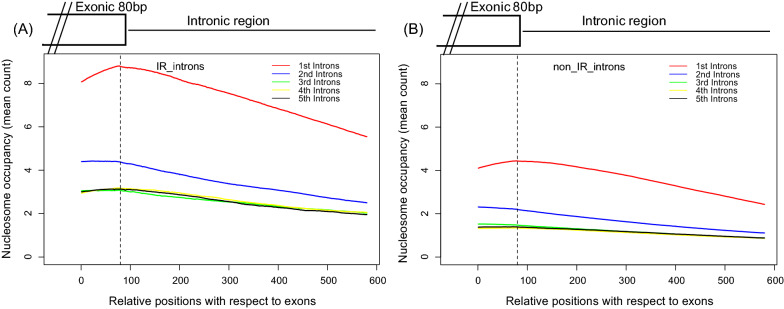
Nucleosome enrichment on the downstream region of exons (intron regions) for **A** retained introns (IR), **B** non-retained introns (non-IR) against the H3K4me3 nucleosome library. The average nucleosome densities over 580 bp downstream of the 1st, 2nd, 3rd, 4th and 5th exons are shown

In our analysis, we considered the intron as being retained if it showed any level of retention. To examine whether the frequency of retained introns is higher for first introns and their effect of nucleosomal density, we extracted the introns exhibiting IR in transcripts and calculated the nucleosomal occupancy (Fig. 5). We found that the first retained introns show a higher density of H3K4me3-marked nucleosomes than other introns. The absence of high nucleosomal density at non-retained introns suggests that the high signal found upstream of second exons (Fig. 2C) is mainly due to intron retention (Fig. 5A).

## Discussion

The preferential positioning of nucleosomes over exons in lncRNA genes, as they are in protein-coding genes, suggests that lncRNA exons are also likely to be subject to exon-specific epigenetic regulation by differential histone modifications. The high signal for H3K36me3 in nucleosomes positioned over mRNA exons in differentiated cell lines (Figs. 2D, 3G) but less pronounced in lncRNA exons (Figs. 2C, 3C) has been attributed to the high stability of nucleosomal positioning within exons of active genes [41, 43] and likely reflects the relatively low (i.e., more restricted) level of expression of lncRNAs.

The low density of H3K36me3-marked nucleosomes in mRNA exons in ES cells (Fig. 3B) contrasts with the strong signal seen in differentiated cells (Figs. 2D, 3D). This finding accords with the previous report that ‘poised’ genes with ‘bivalent’ promoters that are commonly observed in pluripotent cells rarely show H3K36me3 occupancy [65].

The high-frequency of short first introns that are frequently retained in lncRNAs has not hitherto been reported. However, our observations are consistent with a recent report that GC-AG introns with weaker donor and acceptor splice sites, as opposed to more common GT-AG splice sites, are preferentially located in lncRNA first introns of shorter length [61].

We also found that the elevated upstream H3K4me3 signal was stronger in alternatively spliced exons compared to constitutively spliced exons, which is consistent with the high level of alternative splicing observed in lncRNA genes [38] and the recent report of the recruitment of U2 spliceosomal snRNPs [11]. A similar high upstream signal was recently reported for H3K4me3-modified nucleosomes flanking skipped exons [66], which raises the possibility that these signals may be a consequence of the juxtaposition of alternatively spliced exons (and consequent cross-linking during chIP-seq protocols) with the promoter/transcription start site, as previously found [67], which would explain the asymmetry of the signal.

The fact that the slabs of high H3K4me3 occupancy extend over a length of > 1 kb suggests that a number of such marked nucleosomes are clustered together, reminiscent of the somewhat controversial ‘solenoid’ structure reported in early studies of chromatin organization [68, 69]. This phenomenon also suggests that such features and structures are a common feature of lncRNAs, perhaps reflecting the (not mutually exclusive, but general) differences between protein-coding and regulatory RNAs, given that intron retention is strongly associated with cell differentiation [70–73], and/or alternative splicing generally.

## Conclusion

There is widespread intron retention and clustered H3K4me3-marked nucleosomes in short first introns of human long non-coding RNAs, which raises intriguing questions about the relationship of intron retention to lncRNA function and chromatin organization.

## Supplementary Information

**Additional file 1: Figure S1.** Nucleosome enrichment on (A) long non-coding exons and (B) mRNA exons for H3K4me3 nucleosome libraries of B cell, T cell, CD4+ T cell and CDd+ T cell types in comparison to flanking introns. Here, average nucleosome densities over 580 bp upstream and downstream of exons are shown and the middle gap indicates the point of discontinuation between ‘upstream’ and ‘downstream’ data series. The nucleosome density is normalized by the number of exons in each case. **Figure S2.** Density of (A) H3K4me3- and (B) H3K36me3-marked nucleosomes on constitutively spliced (CS) and alternatively spliced (AS) exons in CD4+ T cells. **Figure S3.** Nucleosome enrichment on all long non-coding exons (black line) and long non-coding exons excluding the first and second exons (red line) in comparison to flanking introns in the H3K4me3 histone modification library. **Figure S4.** Nucleosome enrichment on downstream of the (A) 1st long non-coding exons and (B) the 1st protein-coding exons for H3K4me3 nucleosome libraries. The average nucleosome densities over 580 bp downstream of the first exons are shown and the middle gap indicates the point of discontinuation between ‘upstream’ and ‘downstream’ data series. The nucleosome density is normalized by the number of exons. **Figure S5.** Nucleosome enrichment on (A) long non-coding exons and (B) mRNA exons for H3K4me3 nucleosome libraries in comparison to flanking introns as a function of the %GC content in the exonic regions. Here, average nucleosome densities over 580 bp upstream and downstream of exons are shown and the middle gap indicates the point of discontinuation between ‘upstream’ and ‘downstream’ data series. The nucleosome density is normalized by the number of exons in each case. **Figure S6.** Nucleosome enrichment on (A) long non-coding exons and (B) mRNA exons for the RNA PolII nucleosome library in comparison to flanking introns. Here, average nucleosome densities over 580 bp upstream and downstream of exons are shown and the middle gap indicates the point of discontinuation between ‘upstream’ and ‘downstream’ data series. The nucleosome density is normalized by the number of exons in each case. **Figure S7.** Nucleosome enrichment on long non-coding exons for (A) H3K4me3 and (B) H3K36me3 nucleosome libraries of K562 cell line in comparison to flanking introns. Here, average nucleosome densities over 580 bp upstream and downstream of exons are shown and the middle gap indicates the point of discontinuation between ‘upstream’ and ‘downstream’ data series. The nucleosome density is normalized by the number of exons in each case. **Table S1.** Links to the UCSC Genome Browser corresponding to the snapshots in Fig. 4. **Table S2.** Genomic coordinates of 200 randomly selected lncRNA intronic regions across the human genome. 

## Data Availability

All data used herein are publicly available at the designated sites. The scripts used herein for data processing and analysis have been deposited at the Github repository (https://github.com/pdey1/IR-H3K4me3-signal) and are available upon request.
