# Supplementary material for: High frequency of intron retention and clustered H3K4me3-marked nucleosomes in short first introns of human long non-coding RNAs
Source: Epigenetics Chromatin. 2021 Sep 27;14:45. doi: 10.1186/s13072-021-00419-2 (PMC8477579; doi:10.1186/s13072-021-00419-2)
Supplement: Supplementary file 1 — Additional file 1: Figure S1. Nucleosome enrichment on (A) long non-coding exons and (B) mRNA exons for H3K4me3 nucleosome libraries of B cell, T cell, CD4+ T cell and CDd+ T cell types in comparison to flanking introns. Here, average nucleosome densities over 580 bp upstream and downstream of exons are shown and the middle gap indicates the point of discontinuation between ‘upstream’ and ‘downstream’ data series. The nucleosome density is normalized by the number of exons in each case. Figure S2. Density of (A) H3K4me3- and (B) H3K36me3-marked nucleosomes on constitutively spliced (CS) and alternatively spliced (AS) exons in CD4+ T cells. Figure S3. Nucleosome enrichment on all long non-coding exons (black line) and long non-coding exons excluding the first and second exons (red line) in comparison to flanking introns in the H3K4me3 histone modification library. Figure S4. Nucleosome enrichment on downstream of the (A) 1st long non-coding exons and (B) the 1st protein-coding exons for H3K4me3 nucleosome libraries. The average nucleosome densities over 580 bp downstream of the first exons are shown and the middle gap indicates the point of discontinuation between ‘upstream’ and ‘downstream’ data series. The nucleosome density is normalized by the number of exons. Figure S5. Nucleosome enrichment on (A) long non-coding exons and (B) mRNA exons for H3K4me3 nucleosome libraries in comparison to flanking introns as a function of the %GC content in the exonic regions. Here, average nucleosome densities over 580 bp upstream and downstream of exons are shown and the middle gap indicates the point of discontinuation between ‘upstream’ and ‘downstream’ data series. The nucleosome density is normalized by the number of exons in each case. Figure S6. Nucleosome enrichment on (A) long non-coding exons and (B) mRNA exons for the RNA PolII nucleosome library in comparison to flanking introns. Here, average nucleosome densities over 580 bp upstream and downstream of exons are show [file 13072_2021_419_MOESM1_ESM.docx]

EPCH-D-21-00029_R2

**Additional file 1**

**Supplementary Figures:**

**Fig. S1.**

Fig. S1. Nucleosome enrichment on (A) long non-coding exons and (B) mRNA exons for H3K4me3 nucleosome libraries of Bcell, Tcell, CD4+ Tcell and CDd+ T cell types in comparison to flanking introns. Here, average nucleosome densities over 580 bp upstream and downstream of exons are shown and the middle gap indicates the point of discontinuation between ‘upstream’ and ‘downstream’ data series. The nucleosome density is normalized by the number of exons in each case.

**Fig. S2.**

Fig. S2 Density of (A) H3K4me3- and (B) H3K36me3-marked nucleosomes on constitutively spliced (CS) and alternatively spliced (AS) exons in CD4+ T cells.

**Fig. S3.**

Fig. S3. Nucleosome enrichment on all long non-coding exons (black line) and long non-coding exons excluding the first and second exons (red line) in comparison to flanking introns in the H3K4me3 histone modification library.

**Fig. S4.**

Fig. S4. Nucleosome enrichment on downstream of the (A) 1^st^ long non-coding exons and (B) the 1^st^ protein-coding exons for H3K4me3 nucleosome libraries. The average nucleosome densities over 580 bp downstream of the first exons are shown and the middle gap indicates the point of discontinuation between ‘upstream’ and ‘downstream’ data series. The nucleosome density is normalized by the number of exons.

**Fig. S5.**

Fig. S5. Nucleosome enrichment on (A) long non-coding exons and (B) mRNA exons for H3K4me3 nucleosome libraries in comparison to flanking introns as a function of the %GC content in the exonic regions. Here, average nucleosome densities over 580 bp upstream and downstream of exons are shown and the middle gap indicates the point of discontinuation between ‘upstream’ and ‘downstream’ data series. The nucleosome density is normalized by the number of exons in each case.

**Fig. S6.**

Fig. S6. Nucleosome enrichment on (A) long non-coding exons and (B) mRNA exons for the RNA PolII nucleosome library in comparison to flanking introns. Here, average nucleosome densities over 580 bp upstream and downstream of exons are shown and the middle gap indicates the point of discontinuation between ‘upstream’ and ‘downstream’ data series. The nucleosome density is normalized by the number of exons in each case.

**Fig. S7.**

Fig. S7. Nucleosome enrichment on long non-coding exons for (A) H3K4me3 and (B) H3K36me3 nucleosome libraries of K562 cell line in comparison to flanking introns. Here, average nucleosome densities over 580 bp upstream and downstream of exons are shown and the middle gap indicates the point of discontinuation between ‘upstream’ and ‘downstream’ data series. The nucleosome density is normalized by the number of exons in each case.

**Table S1: Links to the UCSC Genome Browser corresponding to the snapshots in Fig. 4:**

|  | **UCSC Genome Browser Links** |
| --- | --- |
| 1 | https://genome.ucsc.edu/cgi-bin/hgTracks?db=hg38&lastVirtModeType=default&lastVirtModeExtraState=&virtModeType=default&virtMode=0&nonVirtPosition=&position=chr12%3A70241000%2D70245000&hgsid=1132772073_TEG8g2nKfinTJaZ8EAjmu3vodyok |
| 2 | https://genome.ucsc.edu/cgi-bin/hgTracks?db=hg38&lastVirtModeType=default&lastVirtModeExtraState=&virtModeType=default&virtMode=0&nonVirtPosition=&position=chr15%3A44534500%2D44539000&hgsid=1132772073_TEG8g2nKfinTJaZ8EAjmu3vodyok |
| 3 | https://genome.ucsc.edu/cgi-bin/hgTracks?db=hg38&lastVirtModeType=default&lastVirtModeExtraState=&virtModeType=default&virtMode=0&nonVirtPosition=&position=chr19%3A40088000%2D40093000&hgsid=1132772073_TEG8g2nKfinTJaZ8EAjmu3vodyok |
| 4 | https://genome.ucsc.edu/cgi-bin/hgTracks?db=hg38&lastVirtModeType=default&lastVirtModeExtraState=&virtModeType=default&virtMode=0&nonVirtPosition=&position=chr7%3A141736000%2D141740000&hgsid=1132772073_TEG8g2nKfinTJaZ8EAjmu3vodyok |

**Table S2: Coordinates of 200 randomly selected lncRNA intronic regions across the human genome:**

| **Coordinates of lncRNA intronic regions** |
| --- |
| Chr1:803067-803647 |
| Chr1:918835-919415 |
| Chr1:1397157-1402385 |
| Chr1:1703915-1704495 |
| Chr1:2556616-2557196 |
| Chr1:3624370-3624950 |
| Chr1:28580566-28581146 |
| Chr1:60952604-60953184 |
| Chr1:70711557-70712137 |
| Chr1:78249684-78250264 |
| Chr1:83424156-83424736 |
| Chr1:89631114-89631694 |
| Chr1:89632129-89632709 |
| Chr1:101349547-10135017 |
| Chr1:103418735-103419315 |
| Chr1:161600740-161601320 |
| Chr1:170234237-170234817 |
| Chr1:177937433-177938013 |
| Chr1:182087771-182088351 |
| Chr1:187268896-187269476 |
| Chr1:187328491-187329071 |
| Chr1:201361942-201362522 |
| Chr1:207867943-207868530 |
| Chr1:213837215-213837795 |
| Chr1:219429095-219429675 |
| Chr1:225710003-225710583 |
| Chr1:239720098-239720680 |
| Chr1:240770583-240771163 |
| Chr1:244010197-244010777 |
| Chr2:6313069-6313649 |
| Chr2:7426351-7426931 |
| Chr2:7887729-7888309 |

| Chr2:10717704-10718284 |
| --- |
| Chr2:19868575-19869155 |
| Chr2:22531025-22531605 |
| Chr2:35163628-35164208 |
| Chr2:60339628-60340208 |
| Chr2:86562346-86562926 |
| Chr2:121649968-121650548 |
| Chr3:48847605-48848185 |
| Chr3:106839512-106840092 |
| Chr3:123585142-123585722 |
| Chr3:184134513-184135093 |
| Chr3:194077901-194078481 |
| Chr4:3917821-3918401 |
| Chr4:9201545-9202125 |
| Chr4:15001645-15002225 |
| Chr4:16425865-16426445 |
| Chr4:76148707-76149287 |
| Chr4:118278444-118279024 |
| Chr5:14663971-14664551 |
| Chr5:77086987-77087567 |
| Chr5:88282510-88283090 |
| Chr5:92507385-92507965 |
| Chr5:180831236-180831816 |
| Chr6:30326183-30326763 |
| Chr6:37533776-37534356 |
| Chr6:50103419-50103999 |
| Chr6:85677795-85678375 |
| Chr6:85677952-85678532 |
| Chr6:85678108-85678688 |
| Chr6:145827861-145828441 |
| Chr6:152805048-152805628 |
| Chr6:159044124-159044704 |
| Chr6:167822690-167823270 |
| Chr7:1582662-1583242 |

| Chr7:8262080-8262660 |
| --- |
| Chr7:9742747-9743327 |
| Chr7:44986017-44986597 |
| Chr7:87345153-87345733 |
| Chr7:112956562-112957142 |
| Chr7:122367570-122368150 |
| Chr7:131106537-131107117 |
| Chr7:131928890-131929470 |
| Chr7:141737487-141738067 |
| Chr7:144207193-144207773 |
| Chr7:150037115-150037695 |
| Chr8:381112-381692 |
| Chr8:1379099-1379679 |
| Chr8:2530081-2530661 |
| Chr8:7325118-7325698 |
| Chr8:9332426-9333006 |
| Chr8:12475457-12476037 |
| Chr8:18084742-18085322 |
| Chr8:22169580-22170160 |
| Chr8:37561339-37561919 |
| Chr8:46848861-46849441 |
| Chr8:60653218-60653798 |
| Chr8:111512547-111513127 |
| Chr8:124474114-124474694 |
| Chr9:3192960-3193540 |
| Chr9:6703953-6704533 |
| Chr9:12146321-12146901 |
| Chr9:22023444-22024024 |
| Chr9:23511688-23512268 |
| Chr9:33799833-33800413 |
| Chr9:37079842-37080422 |
| Chr9:106672369-106672949 |
| Chr9:112486461-112487041 |
| Chr9:117802481-117803061 |

| Chr10:1160053-1160633 |
| --- |
| Chr10:3011090-3011670 |
| Chr10:3502425-3503005 |
| Chr10:4656172-4656752 |
| Chr10:6583618-6584198 |
| Chr10:6920458-6921038 |
| Chr10:10061152-10061732 |
| Chr10:13744013-13744593 |
| Chr10:21368475-21369055 |
| Chr10:33686540-33687120 |
| Chr10:50628531-50629111 |
| Chr10:79808883-79809463 |
| Chr10:95755784-95756364 |
| Chr10:100380716-100381296 |
| Chr10:125707120-125707700 |
| Chr11:4180132-4180712 |
| Chr11:7457863-7458443 |
| Chr11:8013650-8014230 |
| Chr11:9757763-9758343 |
| Chr11:15926732-15927312 |
| Chr11:29350323-29350903 |
| Chr11:41786784-41787364 |
| Chr11:62855136-62855716 |
| Chr11:62855665-62856245 |
| Chr11:66265140-66265720 |
| Chr11:75215170-75215750 |
| Chr11:94679456-94680036 |
| Chr11:123062831-123063411 |
| Chr11:123062831-123063411 |
| Chr12:47209378-47209958 |
| Chr12:52221477-52222057 |
| Chr12:65559472-65560052 |
| Chr12:70242813-70243393 |
| Chr12:92146162-92146742 |

| Chr13:20566612-20567192 |
| --- |
| Chr13:24570063-24570643 |
| Chr13:34534325-34534905 |
| Chr13:46463939-46464519 |
| Chr13:75549476-75550056 |
| Chr14:23712695-23713275 |
| Chr14:23953953-23954533 |
| Chr14:26895746-26896326 |
| Chr14:31262163-31262743 |
| Chr14:95157693-95158273 |
| Chr14:95157693-95158273 |
| Chr15:24546830-24547410 |
| Chr15:36881588-36882168 |
| Chr15:44536278-44536858 |
| Chr15:69091192-69091772 |
| Chr15:74461383-74461963 |
| Chr16:997994-998574 |
| Chr16:3114802-3115382 |
| Chr16:10216375-10216955 |
| Chr16:22374827-22375407 |
| Chr16:30184459-30185039 |
| Chr17:14033581-14034161 |
| Chr17:16439028-16439608 |
| Chr17:20419879-20420459 |
| Chr17:21551036-21551616 |
| Chr17:51323652-51324232 |
| Chr18:31556041-31556621 |
| Chr18:49205956-49206536 |
| Chr19:22022204-22022784 |
| Chr19:31178224-31178804 |
| Chr19:39532616-39533196 |
| Chr19:40090155-40090735 |
| Chr19:46180735-46181315 |
| Chr19:58002711-58003291 |

| Chr19:58359243-58359823 |
| --- |
| Chr19:58569884-58570464 |
| Chr20:1811144-1811724 |
| Chr20:5433882-5434462 |
| Chr20:10231539-10232119 |
| Chr20:18793723-18794303 |
| Chr20:33993070-33993650 |
| Chr20:46474608-46475188 |
| Chr20:49278604-49279184 |
| Chr20:52324962-52325542 |
| Chr20:62430364-62430944 |
| Chr20:64310767-64311347 |
| Chr21:14913442-14914022 |
| Chr21:18034549-18035129 |
| Chr21:22043007-22043587 |
| Chr21:24441295-24441875 |
| Chr21:38120328-38120908 |
| Chr21:44517759-44518339 |
| Chr22:18188576-18189156 |
| Chr22:21172944-21173524 |
| Chr22:25110917-25111497 |
| Chr22:27919223-27919803 |
| Chr22:32362978-32363558 |
| Chr22:42124020-42124600 |
| ChrX:63399823-63400403 |
| ChrX:73944084-73944664 |
| ChrX:73998268-73998848 |
| ChrX:102599206-102599786 |
| ChrX:103919000-103919580 |
| ChrX:155488792-155489372 |
| ChrY:18850825-18851405 |
| ChrY:25730292-25730872 |
